# Applying four-component instructional design to develop a case presentation curriculum

**DOI:** 10.1007/s40037-018-0443-8

**Published:** 2018-07-10

**Authors:** Michelle Daniel, Jennifer Stojan, Margaret Wolff, Bizath Taqui, Tiffany Glasgow, Susan Forster, Todd Cassese

**Affiliations:** 1 Departments of Emergency Medicine and Learning Health Sciences, University of Michigan Medical School, Ann Arbor, MI USA; 2 Departments of Internal Medicine and Paediatrics, University of Michigan Medical School, Ann Arbor, MI USA; 3 Department of Medicine, Lewis Katz School of Medicine, Temple University, Philadelphia, PA USA; 4 Department of Paediatrics, University of Utah School of Medicine, Salt Lake City, UT USA; 5 Department of Ophthalmology and Visual Science, Yale School of Medicine, New Haven, CT USA; 6 Department of Medical Sciences, Frank H. Netter MD School of Medicine, Quinnipiac University, North Haven, CT USA

**Keywords:** Four-component instructional design, Case presentation curriculum, Whole task learning

## Abstract

Medical students must gain proficiency with the complex skill of case presentations, yet current approaches to instruction are fragmented and often informal, resulting in suboptimal transfer of this skill into clinical practice. Whole task approaches to learning have been proposed to teach complex skill development. The authors describe a longitudinal case presentation curriculum developed using a whole task approach known as four-component instructional design (4-C/ID). 4‑C/ID is based on cognitive psychology theory, and carefully attends to titrating a learner’s cognitive load, aiming to always keep students in their zone of proximal development. A multi-institutional group of medical educators convened to develop expert consensus regarding case presentation instruction using the 4‑C/ID model. A curriculum consisting of 1) learning tasks, 2) supportive information, 3) just-in-time information, and 4) part-task practice was developed. Domains were identified that make the task of delivering a case presentation complex. A simplifying conditions approach was applied to each domain to develop sequential task class descriptions. Examples of the four components are given to facilitate understanding of the 4‑C/ID model, making it more accessible to medical educators. Applying 4‑C/ID to curriculum development for the complex skill of case presentation delivery may optimize instruction. The provision of the complete curricular outline may facilitate transfer and implementation of this case presentation curriculum, as well as foster the application of 4‑C/ID to other complex skill development in medical education.

**Electronic supplementary material:**

The online version of this article (10.1007/s40037-018-0443-8) contains supplementary material, which is available to authorized users.

## Introduction

A case presentation is a formal means of communication between health professionals used to convey important clinical information. Components of a full presentation include a patient’s chief concern, history of present illness, relevant past medical, family, and social histories, allergies, medications, a review of systems, the physical exam and an assessment and plan. More abbreviated formats may be used for patient updates and handovers between providers.

The ability to deliver a concise case presentation is a *complex skill* that all medical students must develop to effectively communicate on healthcare teams. The importance of the case presentation is emphasized by its inclusion as the sixth of the Association of American Medical Colleges thirteen Core Entrustable Professional Activities (EPAs) for entering residency: ‘The day 1 resident should be able to concisely present a summary of a clinical encounter to one or more members of the healthcare team in order to achieve a shared understanding of the patient’s current condition’ [1]. Ideally, development of this complex skill occurs longitudinally, beginning in the preclinical years and continuing throughout clinical rotations, yet few longitudinal curricula to teach case presentations have been described [2, 3].

Existing curricula are often fragmented and target particular learner levels in specific contexts [4, 5]. These approaches to teaching case presentations focus learners on sub-components of the larger task, delivered in a series of distinct learning activities, without helping them integrate constituent skills into a meaningful and integrated whole [6]. This creates a ‘transfer paradox’ wherein learners struggle to deliver case presentations in actual clinical environments [7]. A significant body of research within cognitive psychology and educational science suggests whole-task learning may optimize instruction of complex skills, yet this approach has not previously been applied to case presentation instruction [8].

Four-component instructional design (4-C/ID) is a whole task learning approach, developed by Merriënboer and colleagues [7]. 4‑C/ID has been widely applied to curriculum development for various technical skills, but only recently applied to medical education, to develop curricula for communication skills, clinical reasoning, and evidence-based medicine [9–11]. 4‑C/ID, as it is currently presented in the literature [6, 7], can be challenging to comprehend: the diagrams are confusing and complex making the model less accessible to frontline medical educators. For the model to be more broadly implemented, robust and concrete examples are needed to facilitate application. In this paper, we detail the development and content of a case presentation curriculum to serve as a model of 4‑C/ID application in medical education. We describe the challenges we encountered and offer ideas to help other educators translate this important instructional design model into practice.

## The four-component instructional design model

The 4‑C/ID model is designed to carefully titrate cognitive load: Learners progress through a series of whole tasks of increasing difficulty, with each task appropriately scaffolded to maintain learners in their zone of proximal development [12]. The four components of instructional design are 1) *learning tasks*, 2) *supportive information*, 3) *just-in-time information*, and 4) *part-task practice*. The 4‑C/ID model has been described in detail elsewhere [6, 7, 13], but will be briefly recapped here.

*Learning tasks* form the backbone of the curriculum. Learning tasks are based on authentic examples of the whole task (e.g. delivering a case presentation). The learning tasks are organized into ‘task classes,’ deliberately sequenced from simple-to-complex. All the tasks within a given task class are relatively equivalent in complexity. More advanced task classes increase in complexity as the learner demonstrates mastery of a class and moves on to the next. Throughout the curriculum, learning tasks are deliberately varied to provide a wide array of practice opportunities, representative of the variety encountered in real life [7].

A high level of support is provided early on, and progressively diminishes over time, both within and across task classes. This support is labelled as *supportive* or *just-in-time information* depending on whether it supports all of the activities in a given task class or only a specific task. Supportive information is provided at the beginning of a task class and is always available to aid learning. Supportive information can explain how to approach a problem (e.g. offer a cognitive strategy); suggest how the domain is organized (e.g. provide a mental model); or offer foundational knowledge. *Just-in-time information*, in contrast, is only relevant to a specific learning task and not others in the task class. The information is provided right when the learner needs it (e.g. a learner receives a primer on appendicitis before presenting a case of right lower quadrant pain). The final component in the instructional design model is *part-task practice. *It is only needed when a routine aspect of the complex skill requires a degree of automation.

## Approach to developing the curriculum

A multi-institutional group of clinical skills educators convened in 2015 to lay the groundwork for a longitudinal case presentation curriculum using a 4-C/ID approach. Using EPA 6 as a guide, we defined learning objectives [1]. We then set out to identify the constituent components of a case presentation that must be integrated by the learner to meet these objectives. Following an initial brainstorm on what makes delivering a case presentation *complex*, the group organized these ideas into themes with four broad categories: context, public speaking skills, organization, and clinical reasoning (see Fig. 1 of the online electronic supplementary material).

Using the themes and categories as a guide, we developed *task class descriptions* for four task classes of increasing task complexity. All conditions that simplified the whole task of delivering a case presentation were applied to task class 1 (e.g. the context was casual with ample time; notes were permitted; the clinical reasoning was straightforward.) Similar descriptions were developed for the remaining three task classes, with attention given to how complexity increased in each category from task class to task class (see Supplementary Tab. 1–4 of the online electronic supplementary material).

The process of developing the curriculum began once the task class descriptions had been developed. For this step, the authors engaged the broader educational community during a workshop at the 2015 Directors of Clinical Skills Courses (DOCS) Annual Meeting. Twenty-five participants, representing eighteen medical schools, provided input to the curriculum, including curricular deans, course directors and clinical teachers. The core development team collated the ideas, refined the task classes and sent the outlines to workshop attendees for commentary and edits (online Supplementary Tab. 1–4).

## Curricular content

*Learning tasks* were developed, then sequenced across task classes from simple to complex. Within a task class, support for learners was designed to be high initially, then fade over time. The earliest whole tasks were representative of the easiest case presentations a physician might deliver in the real world (e.g. a wrist sprain). Different diseases, settings, and audiences were included over the course of the curriculum to provide variability of practice, to help students develop rich cognitive networks and facilitate transfer into clinical practice.

*Supportive information* was designed to scaffold students’ learning across each task class. Supportive information took many forms, including brief lectures, a data organization exercise, and systematic approaches to problem solving (SAP) that outlined heuristics and rules-of-thumb. The first SAP focused on the content and organization of a case presentation. The second SAP focused on the process of case presentation delivery (e.g. the art of public speaking.) For more advanced task classes, supportive information increased in complexity. For example, the SAP for task class 3 focused more on inclusion of only *relevant* information, *pertinent* positives and negatives, and the development of a more complex assessment and plan.

Each individual learning task was evaluated to determine if *just-in-time information *was valuable to support learning. This was done based on what the average learner at that level would require based on faculty input during the workshop. Not every learning task required this type of scaffolding, but to keep learners in their zone of proximal development, careful attention was paid to titrating intrinsic cognitive load [12]. Intrinsic load was estimated by considering the interaction between the nature of the learning task and the expertise of the learner [14]. One type of *just-in-time information *provided in early task classes was targeted information about diseases. This allowed novice learners to focus on the process and content of case presentation delivery. Another common type of *just-in-time information *was formative feedback. In task class 1, we utilized feedback that shifted emphasis (e.g. feedback focused on organization for task 1.2, feedback focused on public speaking for task 1.3, then feedback focused on both organization and public speaking for task 1.4.) This technique avoids overwhelming the student with too much simultaneous feedback, which can impede learning.

*Part-task practice *was used sparingly, and only when a high level of automaticity was desired to improve performance on routine aspects of the whole task. We used *part-task practice *to teach students to fluently report the review of systems and physical exam findings. In early task classes, students practised reporting ‘normal’ (e.g. regular rate and rhythm, no murmurs rubs or gallops.) We encouraged a thorough approach to reporting early on, to ensure nothing was missed and to familiarize learners with normal terminology. In later task classes, learners needed to become facile with inserting abnormal findings, in addition to determining what was pertinent to include or exclude. Notably, the review of systems and physical exam were always introduced as a component of the whole task first before *part-task practice* was implemented.

## Implementation

The curriculum is intended for longitudinal implementation over several years. Each task class outline contains multiple sessions, and typically aligns with a phase in the curriculum. Tasks are spaced out to provide variable practice over a significant time frame. The total number of curricular hours necessary to implement the curriculum is ~27, counting time to introduce supportive information and complete the 23 learning tasks. This curriculum may be horizontally integrated with instruction of other complex skills in a longitudinal clinical skills course. The number of faculty needed for implementation depends on a school’s pre-existing clinical skills course structure. A typical faculty to learner ratio is 1:8.

A 2–3 h faculty development session on cognitive load and the 4‑C/ID model can help make the curriculum more learner centred. Supportive information, learning tasks and just-in-time information in the outlines are targeted toward the *average* anticipated learner level. Skilled faculty can further adjust the curriculum based on individual learners’ abilities. For instance, a student who is excelling may be given more challenging cases early on, and a learner who is struggling may be offered additional scaffolding. Variations of the curriculum have been implemented at four schools. Most have implemented only Task Classes 1 or 2 to date. Task Classes 3 and 4 will take more time to implement.

## Discussion

Case presentations are complex skills requiring thoughtful instructional design, yet they are often neglected within formal medical school curricula. The authors provide a model curriculum, based on theory, to systematically introduce longitudinal instruction of case presentations. Early introduction of simplified presentation tasks to novice learners prepares students to encounter more challenging learning tasks in the clinical environment where case presentations often significantly impact the impression a student makes on the healthcare team [15], and the quality and safety of patient care [16].

Application of the 4‑C/ID model to case presentation curriculum development has the potential to address several educational obstacles. The first is *compartmentalization*, which occurs when curriculum designers intentionally separate into distinct units the composite knowledge, skills and attitudes necessary for performance of an integrated and complex skill [17]. In our curriculum, knowledge, skills and attitudes are incorporated into each learning task by simultaneously varying the context, case complexity, clinical reasoning, and the audience. The second obstacle is *fragmentation*, which occurs when a complex skill is broken down into all its subcomponents with the expectation that learners will be able to re-assemble those parts at a later point in the performance of the real task [7]. This is addressed in our curriculum by ensuring that even in the earliest task class, learners are confronted with the whole task, but in a manner attending to cognitive load. The third obstacle is the *transfer paradox*, wherein students cannot translate component skills of a complex task into actual practice. Our curriculum addresses this obstacle by ensuring that learners are always engaged in the whole task of delivering a case presentation while moving closer and closer to the highly varied and relatively unpredictable clinical environment.

Some might argue that 4‑C/ID has some features of compartmentalization, fragmentation and the risk of transfer paradox: Supportive and just-in-time information may be introduced in the form of brief lectures, readings or exercises distinct from learning tasks. The backbone of the curriculum, however, centres on learning tasks. This is distinctly different from lecture-based curricula where learners are left to assemble information from disparate sessions into a whole skill. Part-task practice may also seem to isolate components of the whole skill. The use of part-task practice, however, was minimized in our curriculum. When we did utilize it, we always introduced it in the context of the whole task first to provide appropriate context.

To date, the 4‑C/ID model has not been widely implemented in medical education, despite articles that highlight the benefits of whole task learning [6, 8, 17, 18]. Based on our experience, we hypothesize that this lack of adaptation may be multi-factorial: 1) The 4‑C/ID model as currently presented in the literature may be difficult for busy clinician educators to comprehend and apply. The model appears complex when presented in diagrams [6, 7], and may be better understood when concrete examples within medical education are provided. In the words of a member of the development team, ‘When you read about it, you don’t get it. When you sit down with an example applicable to the field you work in and work through it, you get it.’ Ultimately, the fundamental concepts are straightforward: Create curricula comprised of whole learning tasks, sequenced simple-to-complex and scaffold the experience using *supportive information, just-in-time* information and *part-task practice.* If more emphasis were placed on these core and simple to understand principles, perhaps the model would be adapted more broadly. 2) Use of the 4‑C/ID model to teach case presentations challenges educators to depart from traditional teaching of constituent parts towards a whole task approach to foster complex skill development. For implementation to be successful, faculty must understand why whole task approaches are more beneficial to learning. This requires faculty development.

In this paper, we attempt to make the 4‑C/ID model more broadly accessible to medical educators: We present an entire case presentation curriculum outline, with a significant level of detail supported by multiple concrete examples. Such specifics may assist educators considering using the model, not only to implement a case presentation curriculum, but also to design curricula for other complex tasks. We outline a few important steps early in the design process that have not been robustly described elsewhere: The first step involves a collaborative brainstorming session in which instructional designers gather key stakeholders together to identify all aspects of a skill that make it ‘complex.’ The second step involves organizing this brainstorm into a manageable set of themes. Once themes are identified, task class descriptions are developed that address each theme in a developmental manner (online Supplementary Tab. 1–4). This is a replicable process that can be applied to instructional design for other complex skills.

## Caption Electronic Supplementary Material

Supplementary Figure 1: Schematic to represent “what makes a case presentation complex?”

Supplementary Table 1: 4‑C/ID Outline for Task Class 1

Supplementary Table 2: 4‑C/ID Outline for Task Class 2

Supplementary Table 3: 4‑C/ID Outline for Task Class 3

Supplementary Table 4: 4‑C/ID Outline for Task Class 4

## References

[1] Englander R, Aschenbrener C, Call S (2014). Core entrustable professional activities for entering residency curriculum developer’s guide. Association of American Medical Colleges iCollaborative.

[2] Daniel M, Rougas S, Warrier S (2015). Teaching oral presentation skills to second-year medical students. MedEdPORTAL.

[3] Daniel M, Heney R, Kwan B (2015). Preparing for clerkships: learning to deliver specialty-specific oral presentations. MedEdPORTAL.

[4] Heiman HL, Uchida T, Adams C (2012). E-learning and deliberate practice for oral case presentation skills: a randomized trial. Med Teach.

[5] Wiese J, Varosy P, Tierney L (2002). Improving oral presentation skills with a clinical reasoning curriculum: a prospective controlled study. Am J Med.

[6] Vandewaetere M, Manhaeve D, Aertgeerts B, Clarebout G, Van Merriënboer JJ, Roex A (2015). 4C/ID in medical education: how to design an educational program based on whole-task learning: AMEE guide no. 93. Med Teach.

[7] Van Merriënboer JJ, Kirschner PA (2013). Ten steps to complex learning: a systematic approach to four-component instructional design.

[8] Merrill MD (2002). First principles of instruction. Educ Technol Res Dev.

[9] Susilo AP, van Merriënboer J, van Dalen J, Claramita M, Scherpbier A (2013). From lecture to learning tasks: use of the 4C/ID model in a communication skills course in a continuing professional education context. J Contin Educ Nurs.

[10] Postma TC, White JG (2015). Developing clinical reasoning in the classroom–analysis of the 4C/ID-model. Eur J Dent Educ.

[11] Maggio LA, Ten Cate O, Irby DM, O’Brien BC (2015). Designing evidence-based medicine training to optimize the transfer of skills from the classroom to clinical practice: applying the four-component instructional design model. Acad Med.

[12] McLeod SA (2010). Zone of proximal development. Simply psychology.

[13] Van Merriënboer JJ, Clark RE, De Croock MB (2002). Blueprints for complex learning: the 4C/ID-model. Educ Technol Res Dev.

[14] Van Merriënboer JJ, Kester L, Paas F (2006). Teaching complex rather than simple tasks: Balancing intrinsic and germane load to enhance transfer of learning. Appl Cogn Psychol.

[15] Lingard L, Garwood K, Schryer CF, Spafford MM (2003). A certain art of uncertainty: case presentation and the development of professional identity. Soc Sci Med.

[16] Gordon M, Findley R (2011). Educational interventions to improve handover in healthcare: a systematic review. Med Educ.

[17] Dolmans D (2015). When I say … whole-task curricula. Med Educ.

[18] Van Merriënboer JJ, Kester L, Spector JM, Merrill MD, Elen J, Bishop MJ (2007). Whole-task models in education. Handbook of research on educational communications and technology.

