# Supplementary material for: Applying four-component instructional design to develop a case presentation curriculum
Source: Perspect Med Educ. 2018 Jul 10;7(4):276–80. doi: 10.1007/s40037-018-0443-8 (PMC6086819; doi:10.1007/s40037-018-0443-8)
Supplement: Supplementary file 1 — Supplementary Figure 1: Schematic to represent “what makes a case presentation complex?” [file 40037_2018_443_MOESM1_ESM.docx]

**Figure 1:** Schematic to represent “what makes a case presentation complex?”

+/- Positive / Negative; EHR Electronic Health Record; SOAP Subjective Objective Assessment Plan; CC chief concern or complaint; HPI History of Present Illness; PMHx Past Medical History; SHx Social History; FHx Family History; ROS Review of Systems; PE Physical Exam; A/P Assessment and Plan; OPQRST+ Onset of symptom, Palliating / Provocating factors, Quality, Radiation, Severity, Timing, associated symptoms; DDx Differential Diagnosis
