# Supplementary material for: Applying four-component instructional design to develop a case presentation curriculum
Source: Perspect Med Educ. 2018 Jul 10;7(4):276–80. doi: 10.1007/s40037-018-0443-8 (PMC6086819; doi:10.1007/s40037-018-0443-8)
Supplement: Supplementary file 2 — Supplementary Table 1: 4‑C/ID Outline for Task Class 1 [file 40037_2018_443_MOESM2_ESM.docx]

**Table 1: 4-C/ID Outline for Task Class 1**

| **Task Class 1 Description:** | | |
| --- | --- | --- |
| Context:   - Casual, no distractions, classroom or simulated environment - Audience of supportive peers and faculty - Ample time allowed to process information; no time limits on case presentation delivery   Public Speaking Skills:   - Focus on the process of telling a story - Work on a delivery that is confident, natural and engaging - Become cognizant of eye contact, posture, gestures, modulation, fluency and pace, nervous tics and filler words - May rely heavily on notes   Organization:   - Tell a logical, chronologic story of a straightforward case using data only from a single source - Make sure components are generally in the right place (SOAP**^†^**) - Follow a standard internal medicine style format being inclusive of data (pertinent +/- is deferred until later) - Assessment (1 sentence summary), plan (brief)   Clinical Reasoning / Knowledge:   - Simple, involving one problem, few possible systems, leading to a limited differential diagnosis of common diseases - Knowledge of definitions and terms is presumed low - Layman terms are acceptable or definitions are provided - Classic disease representations are used with explanations | | |
| **Supportive Information (given prior to activities)** | | |
| Lecture on case presentation content and process of public speaking  Systematic Approach to Problem Solving: public speaking guidelines  Systematic Approach to Problem Solving: task class 1 case presentation checklist (Appendix 1) with simplified assessment (1-2 sentence summary and brief plan)  SOAP overview and exercise reorganizing mixed up data | | |
| **Learning Task 1.1** | | |
| Watch recorded case presentation of a chest pain case. Analyse using Task Class 1 case presentation checklist and public speaking guidelines. Discuss with peers and faculty in class. | | |
| **Learning Task 1.2** | **Just-in-Time Information** | |
| Read a written interview transcript of a logically organized, simple case (e.g. 6-year old falls out of a tree and injures wrist). Practice telling the story to a peer. | Provide information on wrist fractures versus sprain. Give feedback during activity focused on public speaking skills. | |
| **Learning Task 1.3** | **Just-in-Time Information** | **Part-Task Practice** |
| Watch a video of a well-organized history and physical of a simple case (20-year old with past medical history of asthma presenting with wheezing and shortness of breath). Deliver case presentation to faculty following basic organizational structure of case presentation checklist, considering SOAP. May rely heavily on notes. | Provide information on asthma to aid organization, pulmonary review of systems (ROS^‡^) / physical exam (PE^§^) descriptors. Give feedback focused on case presentation organization. | ROS / PE findings |
| **Learning Task 1.4** | **Just-in-Time Information** | **Part-Task Practice** |
| Interview a standardized patient with dysuria, frequency and low back pain. Deliver case presentation to faculty and peers in simulation centre. | Provide information on differentiating urinary tract infection from pyelonephritis. Give feedback on public speaking skills and organization. | ROS / PE findings |

**†** SOAP Subjective, Objective, Assessment, and Plan; ‡ ROS review of systems; **§** PE physical exam
