# Supplementary material for: Applying four-component instructional design to develop a case presentation curriculum
Source: Perspect Med Educ. 2018 Jul 10;7(4):276–80. doi: 10.1007/s40037-018-0443-8 (PMC6086819; doi:10.1007/s40037-018-0443-8)
Supplement: Supplementary file 3 — Supplementary Table 2: 4‑C/ID Outline for Task Class 2 [file 40037_2018_443_MOESM3_ESM.docx]

**Table 2: 4-C/ID Outline for Task Class 2**

| **Task Class 2 Description:** | | |
| --- | --- | --- |
| Context:   - Low stress, minimal distractions, classroom, simulated or supportive clinical environments - Audience of supportive peers, trainees and faculty - Some time allowed to process information; time for case presentation delivery more limited   Public Speaking Skills:   - Focus on both the content and process of telling a story - Delivery should be confident, natural and engaging - May rely on notes for sections of the presentation   Organization:   - Tell a logical, chronologic story using data from one or more sources for moderately complex cases - Make sure components are in the right place (SOAP^†^) - Follow a standard internal medicine style format, inclusive of relevant data with extraneous data mostly omitted - Assessment includes at least a 3-item differential, justified using data from the history and physical; plan for work-up and treatment plan remains limited   Clinical Reasoning / Knowledge:   - Moderate, involving 1-2 problems, crossing several possible systems, leading to a moderately complex differential diagnosis consisting of mostly common and some rare diseases presenting typically - Knowledge of terms is developing - Layman terms are intermingled with medical terminology - Classic disease representations are used without explanations | | |
| **Supportive Information (given prior to activities)** | | |
| Lecture on how to integrate sources of information in a case presentation  Lecture on clinical reasoning, determining what information to report or exclude in a case presentation  Systematic Approach to Problem Solving: task class 2 case presentation checklist with summary statement, justified differential diagnosis of at least 3 items, and brief plan | | |
| **Learning Task 2.1** | **Just-in-Time Information** | |
| Watch recorded case presentation of a patient with shortness of breath and cough. Multiple sources of data are integrated (patient, significant other, and medical record) into the case presentation. Analyse the case using task class 2 case presentation checklist and public speaking guidelines. Discuss with faculty and peers in class, focusing on data integration and the use of clinical reasoning to construct the case presentation. | Provide information on common causes of shortness of breath and cough (e.g. pneumonia, bronchitis, congestive heart failure, chronic obstructive pulmonary disease exacerbation). | |
| **Learning Task 2.2** | **Just-in-Time Information** | |
| Read a written interview transcript of a logically organized, moderately complex case (e.g. 55-year old with chest pain). The patient is the only source of data. Students have time in advance to prepare to deliver a < 10-minute case presentation to faculty and peers in small groups. Students may rely on notes. | Provide information on causes of chest pain (e.g. acute coronary syndrome, pulmonary embolism, aortic dissection, acid reflux, chest wall pain). Provide feedback focused on organization and the use of clinical reasoning in the case presentation. | |
| **Learning Task 2.3** | **Just-in-Time Information** | **Part-Task Practice** |
| Watch a video of a well-organized history and physical of a moderately complex case (e.g. 50-year old with fever and swelling of the knee). Integrate data from prior medical records (history of sexually transmitted disease). Students have time in advance to prepare to deliver case presentation to faculty and peers in small groups. Students may rely on notes contained on a single index card. | Provide information on causes of fever and mono-articular arthritis (e.g. septic arthritis, crystal disease, Lyme disease). Provide feedback on appropriateness of data included and justified differential diagnosis. | Practice integrating data sources  Practice exercises in determining relevance |
| **Learning Task 2.4** | **Just-in-Time Information** | **Part-Task Practice** |
| Interview a male standardized patient with right lower quadrant abdominal pain. The standardized patient brings past medical record to review. Students have time to prepare an case presentation and record it in the simulation centre for review by faculty. Students may use notes contained on a single index card. | Provide feedback on public speaking skills, organization, and appropriateness of content, as it relates to clinical reasoning, and the assessment and plan. | Practice integrating data sources  Practice exercises in determining relevance |

† SOAP Subjective, Objective, Assessment, and Plan
