# Supplementary material for: Applying four-component instructional design to develop a case presentation curriculum
Source: Perspect Med Educ. 2018 Jul 10;7(4):276–80. doi: 10.1007/s40037-018-0443-8 (PMC6086819; doi:10.1007/s40037-018-0443-8)
Supplement: Supplementary file 4 — Supplementary Table 3: 4‑C/ID Outline for Task Class 3 [file 40037_2018_443_MOESM4_ESM.docx]

**Table 3: 4-C/ID Outline for Task Class 3**

| **Task Class 3 Description:** | |
| --- | --- |
| Context:   - More varied and formal settings - Audience is varied (different specialty and attending preferences) - Time to process information is limited, and time for delivery is constrained (and adjusted by specialty)   Public Speaking Skills:   - Focus on both the content and process of telling a story with a “punch-line” - Polished delivery with minimal use of notes   Organization:   - Tell a logical, chronologic story using data from multiple sources for more complex cases - Follow standard formats as defined by specialty and context - Identify majority of pertinent positives and negatives for inclusion / exclusion - Assessment includes a robust differential diagnosis, justified by data from the history and physical, with some consideration of can’t miss diagnoses; plan for work-up of problems is more complete, and a basic problem-based treatment plan is delineated   Clinical Reasoning / Knowledge:   - Moderate, to complex, involving 1 or more problems, crossing several systems, moderately complex differential diagnosis, consisting of common, rare and can’t miss disease presenting typically or atypically - Knowledge of terms and diseases is moderate - Layman terms are limited. Terminology for review of systems and physical exam descriptors are used consistently. Disease terminology is developing. Begin to “speak the language” of different specialties - Disease representations (illness scripts) are more varied and nuanced | |
| **Supportive Information (given prior to activities)** | |
| Systematic Approach to Problem Solving: task class 3 case presentation checklist (Appendix 2) with summary statement, complete differential diagnosis inclusive of “can’t miss” diseases, justified by data from the history and physical, with a commitment to the most likely diagnosis, and problem-based treatment plan  Lecture on how to adjust a case presentation according to specialty  Systematic Approaches to Problem Solving: core specialty specific case presentation checklists (internal medicine, family medicine, emergency medicine, surgery, obstetrics and gynaecology, paediatrics, psychiatry, neurology) | |
| **Learning Task 3.1** | **Just-in-Time Information** |
| Round robin exercise from MedEdPortal [3]: in small groups, review specialty specific preferences. Read a specialty specific interview transcript. Organize your thoughts for 10 minutes, then pair off and deliver a case presentation following specialty specific guidelines to a peer with minimal reliance on notes. Rotate stations through all 8 core specialties, repeating the process. | May review example videos of specialty specific oral presentations. Feedback is provided by peer using specialty specific case presentation systematic approaches to problem solving. Facilitators float to provide feedback to pairs of students. |
| **Learning Task 3.2** | **Just-in-Time Information** |
| Watch a video of a complex case in which a patient presents to the emergency department with delirium. The patient relates a confusing story. Using a mock electronic medical record, and a phone call to a mock family member, triangulate data sources to deliver a chronologic and well-organized case presentation to faculty in the simulation centre. | Focus feedback on integrating data from multiple sources (the patient was just started on a benzodiazepine and took diphenhydramine according to the spouse). Discuss the differential diagnosis for delirium, and medications that cause it. |
| **Learning Task 3.3** | **Just-in-Time Information** |
| Interview a SP with fever, diffuse abdominal pain, a history of Crohn’s disease on immunosuppressants, and an abscess on Computed Tomography scan. Students have 30 minutes for the interview, and must immediately gather their thoughts and deliver a case presentation to a surgeon. | Feedback is focused on both public speaking skills, and on specialty specific organization of the case. |
| **Learning Task 3.4 - 3.11** | **Just-in-Time Information** |
| Interview a patient selected by the team in each of the 8 core specialties. Students review the medical record and integrate information from other data sources. Students have limited time to prepare the case presentation and present to the team (peers, residents, and / or attending) in a formal clinical setting. Use of notes should be limited to factual data (e.g. vital signs, medications, lab / radiographic results). | Students may look up information on disease presentations as needed or consult the team. Feedback is focused on appropriate use of specialty specific format, use of correct medical terminology, and clinical reasoning as evidenced by what is included / excluded from the case presentation and the assessment and plan. |
