# Supplementary material for: Applying four-component instructional design to develop a case presentation curriculum
Source: Perspect Med Educ. 2018 Jul 10;7(4):276–80. doi: 10.1007/s40037-018-0443-8 (PMC6086819; doi:10.1007/s40037-018-0443-8)
Supplement: Supplementary file 5 — Supplementary Table 4: 4‑C/ID Outline for Task Class 4 [file 40037_2018_443_MOESM5_ESM.docx]

**Table 4: 4-C/ID Outline for Task Class 4**

| **Task Class 4 Description:** | |
| --- | --- |
| Context:   - Dynamic, ever-changing, with distractions and interruptions - Audiences highly varied (attending / specialty preferences, consultants, family-centred rounds, conferences) - Time to process information is limited, time for delivery is constrained and defined by specialties - Patient load increasing (requiring delivery of multiple case presentations at once)   Public Speaking Skills:   - Content and process are seamlessly integrated - Delivery is polished and professional - Notes not needed   Organization:   - Fluidly incorporate data from multiple sources to tell a focused story leading to a clear assessment and plan - Tailor format to specialty and context (admission histories and physicals, SOAP notes, transfers, running the list) - Only pertinent positives and negatives are included with emphasis on relevance and risk factors - Assessment includes a robust differential diagnosis, justified by data from the history and physical, with consideration of “can’t miss” diagnoses and explanation of how excluded, plan for work-up of problems is complete, and a treatment plan is well delineated (problem-based or system-based)   Clinical Reasoning / Knowledge:   - Complex, involving 1 to multiple problems, may cross many or all systems, complex differential diagnoses consisting of common, rare, and can’t miss diseases presenting typically or atypically - Knowledge of terms and diseases is high - “Speaks the language” of different specialties - Disease representations (illness scripts) are robust | |
| **Supportive Information (given prior to activities)** | |
| Lecture on focused case presentations (consultation requests, acutely decompensating patient needing intervention, patient care hand-offs using Situation Background Assessment Recommendation (SBAR)  Discussion: how to quickly adjust the case presentation to audience and setting | |
| **Learning Task 4.1** | **Just-in-Time Information** |
| Read a written interview transcript of a complex case of an 80-year old with multiple co-morbidities and lung cancer, now presenting with signs of spinal cord compression by metastatic disease to the spine. In a simulated environment, consult radiation oncology by phone and deliver a focused case presentation including a targeted question. | Provide feedback on formulating a case presentation to communicate efficiently with a consultant. Discuss the importance of having a targeted clinical question or goal (e.g. seeking palliative radiation, as opposed to calling neurosurgery for spinal cord decompression.) |
| **Learning Task 4.2** | **Just-in-Time Information** |
| After working the overnight shift, practice a hand off of 4 patients to the incoming medical student using SBAR. The team attending observes the transfer of information. An appropriate level of detail should be given for the oncoming student to assume care of the patients. | Focus feedback on use of the SBAR format. Was only critical data included? Was anything pertinent to care omitted? Was the plan going forward clear? |
| **Learning Task 4.3** | **Just-in-Time Information** |
| Interview a patient in the emergency room with undifferentiated shock. Rapidly assess and give a focused case presentation immediately to the attending in < 2 minutes while initiating treatment, in the critical care bay with multiple distractions. Gather additional data from other sources, then call the intensive care unit (ICU) fellow and give a case presentation with the goal of getting the patient admitted. | Discuss different types of shock. Discuss how to nearly simultaneously assess a patient, give a case presentation and initiate treatment. Feedback may be focused on what data to present to convince a consultant (the ICU fellow) to admit the patient. |
| **Learning Task 4.4** | **Just-in-Time Information** |
| Interview a patient and their family in the paediatric ICU. The patient has diabetic ketoacidosis precipitated by pancreatitis, and associated acute renal failure. Give a case presentation with an assessment and plan organized by system to the team at the patient’s bedside with family present. | ICU patient plans are often organized by system. Provide examples of system-based plans for reference. Give feedback focused on family centred communication, content and the organization of the assessment and plan. |
